# Dapsone and N-Acetylcysteine Exert a Synergistic Antinociceptive Effect Associated with Oxidative Stress After Traumatic Spinal Cord Injury: An Isobolographic Study

**DOI:** 10.3390/brainsci16080777

**Published:** 2026-07-23

**Authors:** Héctor Alonso Romero-Sánchez, Camilo Ríos, María de los Ángeles Martínez-Cárdenas, Luz Navarro, Luis Alfonso Moreno-Rocha, Alfonso Mata-Bermudez, Araceli Díaz-Ruiz

**Affiliations:** 1 Maestría y Doctorado en Ciencias Farmacéuticas, Universidad Autónoma Metropolitana Unidad Xochimilco, Mexico City 04960, Mexico; alonsoromero1278@gmail.com; 2 División de Neurociencias, Instituto Nacional de Rehabilitación Luis Guillermo Ibarra Ibarra, Mexico City 14389, Mexico; crios@correo.xoc.uam.mx; 3 Laboratorio de Neurofarmacología Molecular, Departamento de Sistemas Biológicos, Universidad Autónoma Metropolitana, Unidad Xochimilco, Mexico City 04960, Mexico; 4 Departamento de Atención a la Salud, Área de investigación: Estados y Servicios de Salud, Universidad Autónoma Metropolitana Unidad Xochimilco, Mexico City 04960, Mexico; amartinez@correo.xoc.uam.mx; 5 Departamento de Fisiología, Facultad de Medicina, Universidad Nacional Autónoma de México, Mexico City 04360, Mexico; lnavarro@unam.mx; 6 Laboratorio de Farmacología Traslacional, Departamento de Sistemas Biológicos, Universidad Autónoma Metropolitana, Unidad Xochimilco, Mexico City 04960, Mexico; lamoreno@correo.xoc.uam.mx; 7 Centro de Investigación en Ciencias de la Salud (CICSA), FCS (Facultad de Ciencias de la Salud), Universidad Anáhuac México Campus Norte, Huixquilucan 52786, Mexico; 8 Departamento de Neuroquímica, Instituto Nacional de Neurología y Neurocirugía Manuel Velasco Suarez, Mexico City 14269, Mexico

**Keywords:** dapsone, N-acetylcysteine, allodynia, hyperalgesia, oxidative stress, spinal cord injury, isobolographic analysis

## Abstract

**Highlights:**

**What are the main findings?**

- This study demonstrated that dapsone and N-acetylcysteine show an antihyperalgesic effect after traumatic spinal cord injury. Furthermore, both drugs decreased oxidative stress associated with hyperalgesia.
- Combined treatment of dapsone plus N-acetylcysteine showed a synergistic effect on hyperalgesia, demonstrating safety and therapeutic efficacy.

**What are the implications of the main findings?**

- Based on those results, we propose that dapsone plus N-acetylcysteine may be a good combination treatment for pain after spinal cord injury.
- The combination of drugs is novel and opens new therapeutic avenues for research with potential applications in human patients.

**Abstract:**

**Background/Objectives**: Neuropathic pain (NP) after traumatic spinal cord injury (SCI) is highly prevalent, markedly impairs quality of life, and remains difficult to treat because available therapies show limited efficacy and may cause relevant adverse effects. Because oxidative stress and glutamate-mediated excitotoxicity contribute to central sensitization after SCI, this study evaluated the antinociceptive and antioxidant effects of dapsone (DDS), N-acetylcysteine (NAC), and their combination in rats with SCI. An isobolographic analysis was also performed to determine the pharmacodynamic interaction between the two drugs. **Methods**: Female Wistar rats with SCI that developed stimulus-dependent hypersensitivity from day 15 after injury were treated once daily for 7 days with DDS (1.5–12.5 mg/kg, i.p.), NAC (9.3–75 mg/kg, i.p.), or 1:1 fixed-ratio combinations. Mechanical allodynia and hyperalgesia were assessed with Von Frey filaments, whereas lipid peroxidation (LP) and reduced glutathione (GSH) were quantified in injured spinal cord tissue. **Results**: DDS and NAC produced dose-dependent antinociceptive effects, with DDS being more potent than NAC in both endpoints. The individual ED_50_ values were 4.92 and 33.47 mg/kg for allodynia, and 4.06 and 27.74 mg/kg for hyperalgesia, for DDS and NAC, respectively. The DDS/NAC combination showed synergistic interactions in allodynia (ED_50_ = 7.45 mg/kg vs. Zadd = 19.19 mg/kg; γ = 0.388) and hyperalgesia (ED_50_ = 4.21 mg/kg vs. Zadd = 15.90 mg/kg; γ = 0.265), with stronger synergism in hyperalgesia. Combined treatment also improved LP and GSH levels. **Conclusions**: These findings indicate that DDS and NAC exert complementary actions and that their combination produces a synergistic antinociceptive effect associated with diminished oxidative stress after SCI.

## 1. Introduction

NP is among the most severe, persistent, and disabling nonmotor symptoms that develop after SCI, significantly affecting patients’ quality of life [1,2]. NP after SCI arises as a consequence of dysfunction in the somatosensory nervous system [3]. In general, pain may be located above, at the level of, or below the lesion, and it can appear immediately after the acute injury caused by external mechanical forces or develop and increase in intensity years later as a result of molecular cascades and degeneration of neuronal circuits [4]. The prevalence of NP in patients with SCI ranges from 53% to 80%, with the main clinical manifestations including spontaneous pain, which may be continuous or intermittent, abnormal sensations such as burning, tingling, or stabbing pain, and stimulus-dependent hypersensitivity, including allodynia and hyperalgesia, predominantly in the lower extremities [5,6]. Several studies suggest that oxidative stress and increased NMDA receptor excitability, resulting in glutamate-mediated excitotoxicity, are closely related to central sensitization, which is considered a key pathophysiological mechanism underlying NP after traumatic SCI [7,8]. Current pharmacological treatment for NP associated with SCI includes gabapentinoids (gabapentin and pregabalin), tricyclic antidepressants (amitriptyline), opioid analgesics (tramadol and oxycodone), and anticonvulsants (lamotrigine) [9]. However, despite therapeutic advances, no available treatment has yet proven completely effective in restoring sensory function, which supports the need to explore new therapeutic strategies aimed at improving efficacy while reducing the adverse effects of conventional therapies.

In this context, agents with antioxidant and neuroprotective properties have shown promise in reducing pain-related behaviors in animal models, as is the case for dapsone (DDS), a bacteriostatic drug originally indicated for the treatment of leprosy [10]. DDS has been reported to decrease painful sensations such as allodynia and hyperalgesia, probably because of its neuroprotective action through the inhibition of excitotoxicity mediated by NMDA and AMPA/kainate receptors [11,12]. In addition, its antioxidant effects after SCI include decreased lipid peroxidation (LP) and increased reduced glutathione (GSH) levels in rats with NP induced either by SCI or by vincristine administration, further supporting a close relationship between central sensitization and oxidative stress [11,13]. Likewise, N-acetylcysteine (NAC), which is commonly used as a mucolytic agent and as a treatment for paracetamol (acetaminophen) poisoning, has been shown to attenuate NP in rats through modulation of oxidative stress, reduction in nitric oxide (NO), and inhibition of matrix metalloproteinase-9 [14,15,16]. Taken together, these findings indicate that both DDS and NAC, when administered independently, are promising candidates for the treatment of NP resulting from SCI. Nevertheless, combining drugs with different but potentially complementary mechanisms of action may enhance antinociceptive efficacy while reducing the adverse effects associated with monotherapy. Therefore, the present study aimed to evaluate the antinociceptive and antioxidant effects of DDS, NAC, and their combination in animals with SCI.

Specifically, individual dose–response relationships were characterized in mechanical allodynia and hyperalgesia, and an isobolographic analysis was performed to determine whether the pharmacodynamic interaction between DDS and NAC was additive or synergistic, as well as to examine whether these effects were associated with changes in oxidative stress biomarkers in injured spinal cord tissue.

## 2. Materials and Methods

### 2.1. Animals

One hundred and thirty-two adult Wistar rats, weighing approximately 200–250 g, were used, following the standardized methods described for female rats [17]. They were housed in transparent acrylic boxes measuring approximately 49 cm × 34 cm × 16 cm (6 animals per box) with sterile sawdust and free access to food or water. All the animals were housed in a room under standard laboratory conditions (12:12 light-dark cycle, 22 °C). The study subjects were provided by the Animal Production Unit of the National Institute of Neurology and Neurosurgery Manuel Velasco Suárez (Protocol No. 106/2023), approved by the members of the institutional committee for the care and use of laboratory animals, following the guidelines indicated in the Mexican Official Standard NOM-062-ZOO-1999, the Guide for the Care and Use of Laboratory Animals for National Health Institutions (USA) and the ethical standards for pain research in animals of the International Association for the Study of Pain [18] and approved by the scientific committee.

This study was conducted in accordance with the ARRIVE (Animal Research: Reporting of In Vivo Experiments) guidelines (version 2.0) [19]. The completed ARRIVE checklist is provided as (Supplementary Table S1).

### 2.2. Surgical Procedure

Under anesthesia with sodium pentobarbital (50 mg/kg i.p.) followed by asepsis in the thoracic region with iodine alcohol, the animals underwent either a laminectomy (sham surgical procedure) or a contusion SCI according to a previously reported procedure [17]. This procedure involves making an incision from the mid-thoracic region to the lower part of the rat, followed by a laminectomy at the level of the twelfth thoracic vertebra (T-12) to expose the nerve tissue located in the spinal canal. Subsequently, using the impactor device from New York University (NYU), a 10 g metal cylinder circular impact rod with a diameter of 2.0 mm was dropped from a height of 6.25 mm (free fall) directly onto the exposed spinal cord to produce a controlled SCI. Once the damage was verified by the presence of a dorsal hematoma resulting from the rupture of the dorsal spinal artery caused by contusion damage, it was treated at discontinuous points of the muscle tissue and, subsequently, the animal’s skin. Similarly, the animals received postoperative care until complete recovery from anesthesia in a small animal intensive care unit (Schroer Manufacturing Co., Kansas City, KS, USA), and they also received 0.2 mL of benzathine penicillin intramuscularly as a single-dose antibiotic treatment and paracetamol as an analgesic and antipyretic in the drinking water at a dosage of 200 mg/kg for 7 days.

### 2.3. Pharmacological Treatment

DDS, NAC, and gabapentin were obtained from Sigma-Aldrich (St. Louis, MO, USA). For intraperitoneal (i.p.) administration, DDS was dissolved in a solution composed of 15% ethanol, 40% propylene glycol, and 0.9% physiological saline. Dosages of 1.5, 3.1, 6.25, and 12.5 mg/kg i.p. of DDS were used as previously reported [11]. NAC was dissolved in 0.9% physiological saline (0.9% SSF). Doses of 9.3, 18.5, 37.5, and 75 mg/kg i.p. were used, as previously reported [15,20].

### 2.4. Behavioral Tests

The 50% paw withdrawal threshold stimulus-dependent hypersensitivity (tactile allodynia) was evaluated by measuring the 50% paw withdrawal threshold (Up-Down) in response to mechanical stimulation with Von Frey filaments (Stoelting Co., Wood Dale, IL, USA, Semmes-Weinstein Von Frey Aesthesiometer for touch assessment), using a method previously described [21,22] and later modified [23]. For this procedure, each rat was placed in a transparent acrylic chamber on a wire mesh surface for 30 min before each evaluation, allowing the animals to acclimate to the testing environment. Von Frey filaments (with forces of 1.4, 2, 4, 6, 8, 10, and 15 g) were applied vertically for 10 s at one-minute intervals to the plantar surface of the hind paws (right and left). A response was considered positive when there was an abrupt withdrawal of the limb as a result of applying the monofilament. The values obtained from this test were used to calculate the 50% paw withdrawal threshold using the following formula:

$$50\%\;Umbral=\left(\frac{10^{\left(Xf+k\delta \right)}}{10000}\right)$$

where Xf is the value in logarithmic units of the last Von Frey filament used; **k** is the value obtained from the pattern of positive and negative responses [23]; and **δ** is the mean of the differences between the stimuli in logarithmic units.

### 2.5. Assessment of the Average Paw Withdrawal Frequency

The assessment of the average withdrawal frequency of the hind paws in response to a noxious stimulus (mechanical hyperalgesia) was carried out according to a previously described method [24]. To perform this assessment, the animals were placed in observation chambers 30 min before the behavioral evaluation to allow them to adapt to the environment. A Von Frey filament exerting a 15 g (noxious stimulus) was used, which was applied 10 times at approximately 3 s intervals on the plantar surface of the rat’s hind paws (right and left, three different trials), to determine the average paw withdrawal frequency. Animals that did not develop hyperalgesia behavior were excluded from the study.

### 2.6. Lipid Peroxidation Assay

Once the behavioral experiments were completed (21 days post-SCI), the concentrations of LP present in the injured tissue were quantified using a technique previously described by Triggs and Willmore [25] and later modified by Diaz-Ruiz et al. [26]. Samples of naïve, sham, and injured spinal cord tissue (centered at the lesion epicenter) were obtained at the level of the twelfth thoracic vertebra of each rat, approximately 6 mm in length, 3 mm from the caudal area, and 3 mm from the rostral area, with a wet weight between 0.03 and 0.032 g. Subsequently, each tissue fragment was homogenized in 3 mL of 0.9% SSF, and two 1 mL aliquots were separated for the LP assay. The homogenized tissue was added to 4 mL of a chloroform–methanol mixture (2:1, *v*/*v*). The remaining 1 mL aliquot of homogenate was used to measure GSH. The mixture was stirred with the aid of a variable-speed vortex mixer and cooled with ice for 30 min to allow phase separation between the chloroform and methanol. The fluorescence of the final stable LP products contained in the chloroform phase was determined using a Perkin-Elmer LS50B luminescence spectrophotometer at 370 nm (excitation) and 430 nm (emission). The sensitivity of the spectrophotometer was set to 150 fluorescence units using a standard quinine solution (0.1 mg/mL). The results are expressed as international fluorescence units per gram of wet tissue.

### 2.7. Determination of the Reduced Glutathione Concentrations

The concentration of GSH in the medullary tissue of rats subjected to SCI or a sham surgical procedure was determined using the method described by Hissin and Hilf [27]. For this determination, a GSH standard dissolved in a 0.1 M sodium phosphate buffer solution with 5 mM EDTA (pH 8) was used. The tissue samples were homogenized in 3.75 mL of 0.1 M sodium phosphate buffer solution with 5 mM EDTA (pH 8) and 1 mL of 25% HPO_3_. They were then centrifuged at 3000× *g* for 15 min. Five hundred microliters of the obtained supernatants were added to 4.5 mL of the phosphate buffer solution, plus 100 µL of o-phthalaldehyde. The mixtures were incubated at room temperature for 15 min, and the fluorescence signals were measured using a Perkin Elmer LS50B luminescence spectrophotometer at 350 nm (excitation wavelength) and 420 nm (emission wavelength). The results are expressed as µmol of GSH per g of wet tissue (spinal cord) according to the report by Diaz-Ruiz et al. [28].

### 2.8. Evaluation of the Antinociceptive Effect Induced by Subacute Treatment with DDS or NAC in Rats with SCI

**Figure 1:**
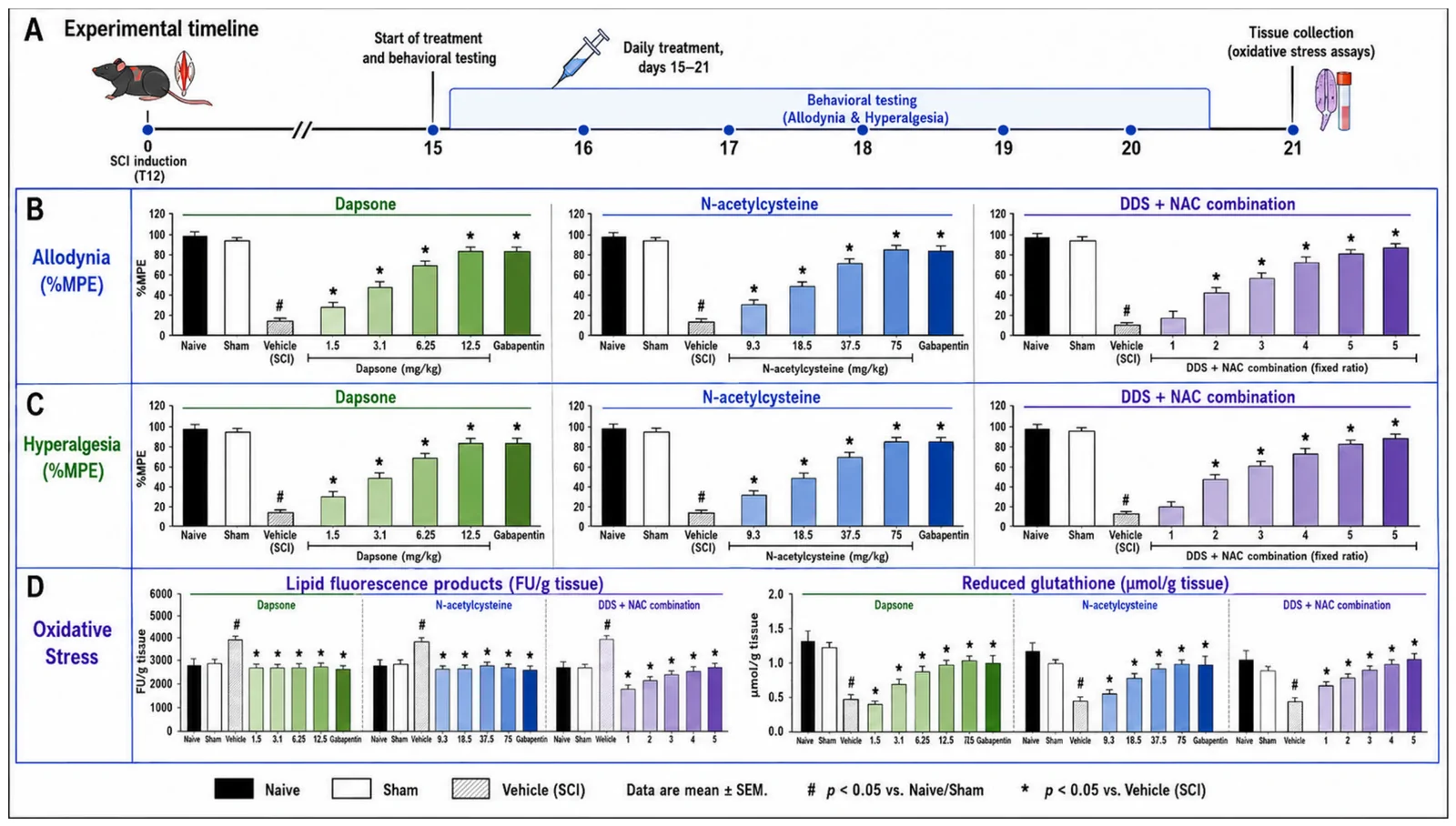
Experimental timeline and effects of dapsone, N-acetylcysteine, and their combination after spinal cord injury. (**A**) Experimental timeline showing SCI induction at T12, treatment, and behavioral testing from days 15–21, and tissue collection on day 21. (**B**,**C**) Effects of subacute dapsone (DDS), N-acetylcysteine (NAC), and the fixed-ratio DDS + NAC combination on tactile allodynia and mechanical hyperalgesia, respectively. Within each panel, DDS, NAC, and combination results are shown from left to right. Behavioral responses were assessed every 24 h in both hind paws using Von Frey filaments. Bar graphs show %MPE values calculated from the area under the curve of the 8 h assessments. (**D**) Lipid fluorescence products and reduced glutathione (GSH) levels in spinal cord tissue; green, blue, and magenta graphs correspond to DDS, NAC, and DDS + NAC groups, respectively. Combination groups 1–5 represent DDS + NAC doses of 0.43 + 2.75, 0.76 + 4.90, 2.41 + 11.49, 7.63 + 48.99, and 13.58 + 87.12 mg/kg. Data are mean ± SEM of 4–7 animals per group. One-way ANOVA followed by Tukey’s test; # *p* < 0.05 versus naïve/sham and * *p* < 0.05 versus SCI + vehicle. SCI, spinal cord injury; FU, fluorescence units; %MPE, percentage of maximum possible antinociceptive effect.

Before SCI was induced by contusion at the level of the twelfth thoracic vertebra (T12) with mild intensity by dropping a metal cylinder from a height of 6.25 mm, baseline values of the 50% paw withdrawal threshold (allodynia) and the mean frequency of hind paw withdrawal in response to a noxious stimulus (hyperalgesia) were obtained. To evaluate the possible antinociceptive effect of subacute treatment (7 days) with DDS or NAC 15 days after injury, 84 animals weighing approximately 200 to 250 g were randomly distributed into 14 experimental groups (*n* = 6), with the help of a pocket calculator. The sample size for each group was calculated using the following formula:

$$n=2{\left[\frac{\left(Z\alpha +z\beta \right)\sigma }{\mu_{1}-\mu_{2}}\right]}^{2}$$

where *n* is the number of subjects for each treatment group, *μ*_1_–*μ*_2_ is the detectable difference between the means of the two groups, *σ* is the weighted standard deviation (Sp) of each group, and *Zα* and *Zβ* are the values that include *α* in both tails and *β* in the lower tail of the standard normal distribution obtained from the *Z* tables of Dawson [29]. Likewise, the first group did not receive pharmacological treatment (Naïve). The second group consisted of rats that underwent only a simulated surgical procedure (Sham). The third and fourth groups received SCI plus dapsone and NAC vehicles (0.9% NaCl), respectively, for 7 days and then administered every 24 h. From the fifth group to the twelfth group with SCI, they received increasing doses of DDS (1.5, 3.1, 6.25, and 12.5 mg/kg, i.p., for 7 days every 24 h) or NAC (9.3, 18.5, 37.5, and 75 mg/kg, i.p., every 24 h for 7 days) (Table 1). The remaining SCI group received gabapentin as a positive control (30 mg/kg, i.p. for 7 days every 24 h) 10 min before stimulus-dependent hypersensitivity (allodynia and hyperalgesia) was assessed with Von Frey filaments. At the end of the experiments, the animals were sacrificed by decapitation (after the administration of a sodium pentobarbital overdose). Immediately, a portion of the injured tissue was extracted to determine the concentrations of LP and GSH present in the injured tissue. Finally, both the behavioral assessment and the biochemical assays were performed blinded to the treatment (see Figure 1A).

### 2.9. Calculation of the Percentage of Maximal Possible Effect

To compare allodynic and hyperalgesic responses between treatment groups, effects were normalized as the percentage of maximal possible effect (%MPE), calculated from the area under the curve (AUC) of behavioral time courses using 50% paw withdrawal threshold (allodynia) and mean hind paw withdrawal frequency (hyperalgesia), with the following equation:

$$\%MPE=\left(\frac{\left(AUC_{compound})-(AUC_{vehicle}\right)}{\left(AUC_{sham})-(AUC_{vehicle}\right)}\right)\times 100$$

where $AUC_{compound}$ is the area under the curve obtained for the drug-treated group, $AUC_{vehicle}$ is the area under the curve of the vehicle-treated SCI group, and $AUC_{sham}$ is the area under the curve of the sham group. This transformation was used to normalize all responses and was subsequently applied to the construction of individual and mixture dose–response curves.

### 2.10. Pharmacodynamic and Isobolographic Analysis of DDS/NAC Combination

The antinociceptive effects of DDS, NAC, and their combination were evaluated in experimental models of mechanical allodynia and hyperalgesia. For each treatment condition, the response was expressed as %MPE. Individual dose–response data for DDS and NAC were first analyzed separately in order to characterize their pharmacodynamic profiles and to derive the parameters required for the subsequent interaction analysis. In addition, the experimental combination groups were analyzed at the individual-animal level to assess dose-related effects and to estimate the uncertainty associated with the pharmacometric parameters of the mixture.

Dose–response relationships were analyzed by nonlinear regression using a sigmoidal Emax/Hill model according to the following equation:

$$E(D)=\frac{E_{max}\cdot D^{h}}{ED_{50}^{h}+D^{h}}$$

where E(D) is the predicted effect at dose D, $E_{max}$ is the maximal effect, $ED_{50}$ is the dose producing 50% of the maximal effect, and h is the Hill coefficient. Because the outcome variable was expressed as %MPE, the maximal response was normalized to 100. Therefore, the principal free parameters estimated by the model were $ED_{50}$ and the Hill slope. Nonlinear fitting was performed by least-squares minimization of the residuals between observed and predicted values. Model performance was evaluated by visual inspection of the fitted curves together with the experimental data and by calculation of the apparent coefficient of determination ($R^{2}$) and the root mean square error (RMSE). This approach is consistent with standard quantitative pharmacological analyses of dose–effect relationships and with the derivation of $ED_{50}$ values for subsequent interaction studies [30,31].

The DDS/NAC combinations were evaluated using a fixed equipotent ratio. In this design, each drug contributed the same fraction of its respective ED_50_ rather than the same absolute dose in mg/kg. The experimental mixture doses were selected prospectively using the preliminary individual ED_50_ estimates available when the combination study was designed. Both drug components were multiplied by the same scaling factor, thereby preserving their relative equipotent contribution while generating a sufficiently broad dose range to characterize the ascending and maximal portions of the mixture dose–response curve. After completion of the study, nonlinear regression of the complete individual-drug datasets yielded final ED_50_ values of 4.92 mg/kg for DDS and 33.46 mg/kg for NAC. Accordingly, the definitive theoretical additive dose corresponding to an equipotent 1:1 combination was calculated as one-half of the ED_50_ of each compound:

$$Z_{add}=\frac{4.92}{2}+\frac{33.46}{2}=2.46\frac{mg}{kg}DDS+16.73\frac{mg}{kg}NAC=19.19\;mg/kg$$

Thus, the term 1:1 refers to equal fractions of the individual ED_50_ values and not to equal absolute doses. The corresponding DDS:NAC mass ratio was approximately 1:6.80. The final ED_50_ values were used for the definitive isobolographic analysis and calculation of the interaction index.

The combination doses evaluated were 0.43 mg/kg DDS + 2.75 mg/kg NAC, 0.76 mg/kg DDS + 4.90 mg/kg NAC, 2.41 mg/kg DDS + 11.49 mg/kg NAC, 7.63 mg/kg DDS + 48.99 mg/kg NAC, and 13.58 mg/kg DDS + 87.12 mg/kg NAC, corresponding to total doses of 3.18, 5.66, 13.90, 56.62, and 100.70 mg/kg, respectively. These combinations were used to construct the mixture of dose–response curves for both allodynia and hyperalgesia (Table 1).

For the mixture analysis, nonlinear regression was performed using the total mixture dose as the independent variable [30,32]. Drug interaction was assessed by isobolographic analysis by comparing the experimental mixture $ED_{50}$($Z_{mix}$) with the theoretical additive dose according to the following equation:

$$\gamma =\frac{Z_{mix}}{Z_{add}}$$

Values of γ<1 were interpreted as synergism, values close to 1 as additivity, and values of γ>1 as antagonism. A potency shift was also calculated as $Z_{add}/Z_{mix}$, where Z_add_ is the total theoretical additive dose of the combination, and Z_mix_ is the actual total experimental dose of the mixture that produced the effect.

For graphical representation, the experimental mixture $ED_{50}$ was projected onto the theoretical equipotent ratio and plotted on an isobologram together with the line of additivity connecting the individual $ED_{50}$ values of DDS and NAC.

### 2.11. Statistical Analysis

Data are presented as mean ± S.E.M. Behavioral and biochemical results from all groups were analyzed using one-way ANOVA, with Tukey’s test applied when appropriate (significance set at *p* < 0.05). Analyses were performed using SPSS Version 25.0 (IBM Corp., 2017 Armonk, New York, NY, USA). The Kolmogorov–Smirnov and Levene’s tests assessed normality and homogeneity of variance to determine use of parametric or nonparametric statistics. One-way ANOVA was also used to analyze the area under the curve (AUC) of the behavioral time courses for each treatment.

For pharmacodynamic analyses, individual and mixture dose–response curves were fitted by nonlinear regression to a sigmoidal Emax/Hill model. From these fits, the main pharmacometric parameters were estimated, including $ED_{20}$, $ED_{50}$, $ED_{80}$, $ED_{90}$, and the Hill slope. Model performance was assessed by the apparent coefficient of determination ($R^{2}$) and the root mean square error (RMSE).

The uncertainty of the pharmacometric parameters of the mixture was estimated by stratified bootstrap resampling by dose level. For each bootstrap replicate, individual-animal values within each dose were resampled with replacement, group means were recalculated, and the mixture dose–response curve was refitted. The resulting distributions were used to estimate median values and 95% confidence intervals for $ED_{20}$, $ED_{50}$, $ED_{80}$, $ED_{90}$, the Hill slope, and the interaction index γ. This resampling procedure was implemented in R (^©^The R Foundation for Statistical Computing, Vienna, Austria) using custom scripts for nonlinear model refitting and bootstrap estimation.

## 3. Results

The experimental sequence is summarized in Figure 1A. Comparative effects of dap-sone (DDS), N-acetylcysteine (NAC), and their fixed-ratio combination on mechanical allodynia, mechanical hyperalgesia, and spinal cord oxidative stress markers are presented in Figure 1B–D.

### 3.1. Subacute Treatment with DDS Reduces Stimulus-Dependent Hypersensitivity and Oxidative Stress in Rats with SCI

Subacute DDS administration (1.5, 3.1, 6.25, and 12.5 mg/kg, i.p., once daily for 7 days) produced dose-dependent antinociceptive effects (Figure 1B,C, left panels). Naïve and sham animals maintained a 50% paw withdrawal threshold of approximately 14 g and a hind paw withdrawal frequency of approximately 1, indicating the absence of evoked nociceptive behavior. In SCI animals, DDS increased the withdrawal threshold and reduced withdrawal responses from the first administration (day 15 post-SCI) through day 21. At 12.5 mg/kg, DDS produced 92.65% MPE for allodynia and 92.46% MPE for hyperalgesia.

DDS also attenuated oxidative stress in injured spinal cord tissue (Figure 1D, green bars). Lipid fluorescence products were 2732.90 ± 73.06 and 2760.78 ± 82.19 FU/g tissue in the naïve and sham groups, respectively, and increased to 3964.90 ± 262.46 FU/g tissue in the SCI + vehicle group. DDS at 3.1 and 12.5 mg/kg reduced lipid fluorescence products by 19.78% and 19.65%, respectively, relative to vehicle; gabapentin reduced them by 17.19%. GSH concentrations were 1.18 ± 0.09 and 1.06 ± 0.04 µmol/g tissue in the naïve and sham groups, respectively, and 0.50 ± 0.04 µmol/g tissue in the SCI + vehicle group. DDS significantly increased GSH at 6.25 and 12.5 mg/kg (0.89 ± 0.05 and 0.89 ± 0.06 µmol/g tissue, respectively).

### 3.2. Subacute Treatment with NAC Reduces Stimulus-Dependent Hypersensitivity and Oxidative Stress in Rats with SCI

Subacute NAC administration (9.3, 18.5, 37.5, and 75 mg/kg, i.p., once daily for 7 days) also produced dose-dependent antinociceptive effects (Figure 1B,C, middle panels). The increase in the 50% paw withdrawal threshold was evident from day 16 post-SCI and persisted through day 21, while hind paw withdrawal responses decreased toward the values observed in naïve and sham animals. At 75 mg/kg, NAC produced 87.88% MPE in both behavioral endpoints.

NAC significantly reduced lipid fluorescence products at 18.5, 37.5, and 75 mg/kg by 22.79%, 26.70%, and 28.60%, respectively, with corresponding values of 3002.09 ± 106.54, 2849.99 ± 48.92, and 2776.23 ± 23.81 FU/g tissue, compared with 3888.34 ± 80.52 FU/g tissue in the SCI + vehicle group (Figure 1D, blue bars). No significant reduction was observed at 9.3 mg/kg, whereas gabapentin reduced lipid fluorescence products by 16%. GSH concentrations in the NAC groups were 0.51 ± 0.05, 0.71 ± 0.04, 0.89 ± 0.07, and 0.85 ± 0.10 µmol/g tissue at 9.3, 18.5, 37.5, and 75 mg/kg, respectively; gabapentin yielded 0.89 ± 0.05 µmol/g tissue. Significant increases relative to the SCI + vehicle group (0.53 ± 0.06 µmol/g tissue) were observed at the higher NAC doses.

### 3.3. Subacute Treatment with a Combination of DDS and NAC Reduced Stimulus-Dependent Hypersensitivity and Oxidative Stress in Rats with SCI

The fixed-ratio DDS + NAC combinations listed in Table 1 produced dose-dependent antiallodynic and antihyperalgesic effects (Figure 1B,C, right panels). The highest combination (13.58 mg/kg DDS + 87.12 mg/kg NAC) produced 99.14% MPE for allodynia and 105.03% MPE for hyperalgesia. The pharmacometrics characterization of the individual compounds and the combination is presented in Section 3.4.

Combined treatment also modified both oxidative stress markers (Figure 1D, magenta bars). Lipid fluorescence products were 2249.97 ± 112.38 and 2820.65 ± 207.93 FU/g tissue in the naïve and sham groups, respectively, and increased to 4746.27 ± 226.21 FU/g tissue in the SCI + vehicle group. Combination groups 1–5 yielded 3544.78 ± 148.85, 3540.14 ± 107.90, 3030.24 ± 95.39, 3488.69 ± 48.99, and 2459.55 ± 93.68 FU/g tissue, respectively. GSH concentrations were 1.08 ± 0.06 µmol/g tissue in naïve animals, 0.84 ± 0.07 µmol/g tissue in sham animals, and 0.51 ± 0.03 µmol/g tissue in the SCI + vehicle group. The corresponding values for combination groups 1–5 were 0.82 ± 0.06, 0.80 ± 0.05, 0.91 ± 0.07, 1.00 ± 0.11, and 1.03 ± 0.07 µmol/g tissue. Except for combination 2, the increases in GSH were significant relative to the vehicle, and the two highest combinations restored GSH toward basal values.

To further characterize the pharmacodynamic interaction underlying these behavioral effects, nonlinear dose–response modeling and isobolographic analyses were subsequently performed.

### 3.4. Pharmacodynamic and Isobolographic Analysis of the DDS/NAC Combination Demonstrates Synergistic Antinociceptive Interactions in Rats with SCI

DDS and NAC showed clear sigmoidal dose–response relationships in both allodynia and hyperalgesia, consistent with the Emax/Hill model. In allodynia, the dose–effect relationship for DDS was described by:

$$E_{DDS-Allo}=\frac{100\cdot D^{2.642}}{4.92^{2.642}+D^{2.642}}$$

whereas the NAC curve was fitted by:

$$E_{NAC-Allo}=\frac{100\cdot D^{2.721}}{33.46^{2.721}+D^{2.721}}$$

**Figure 2:**
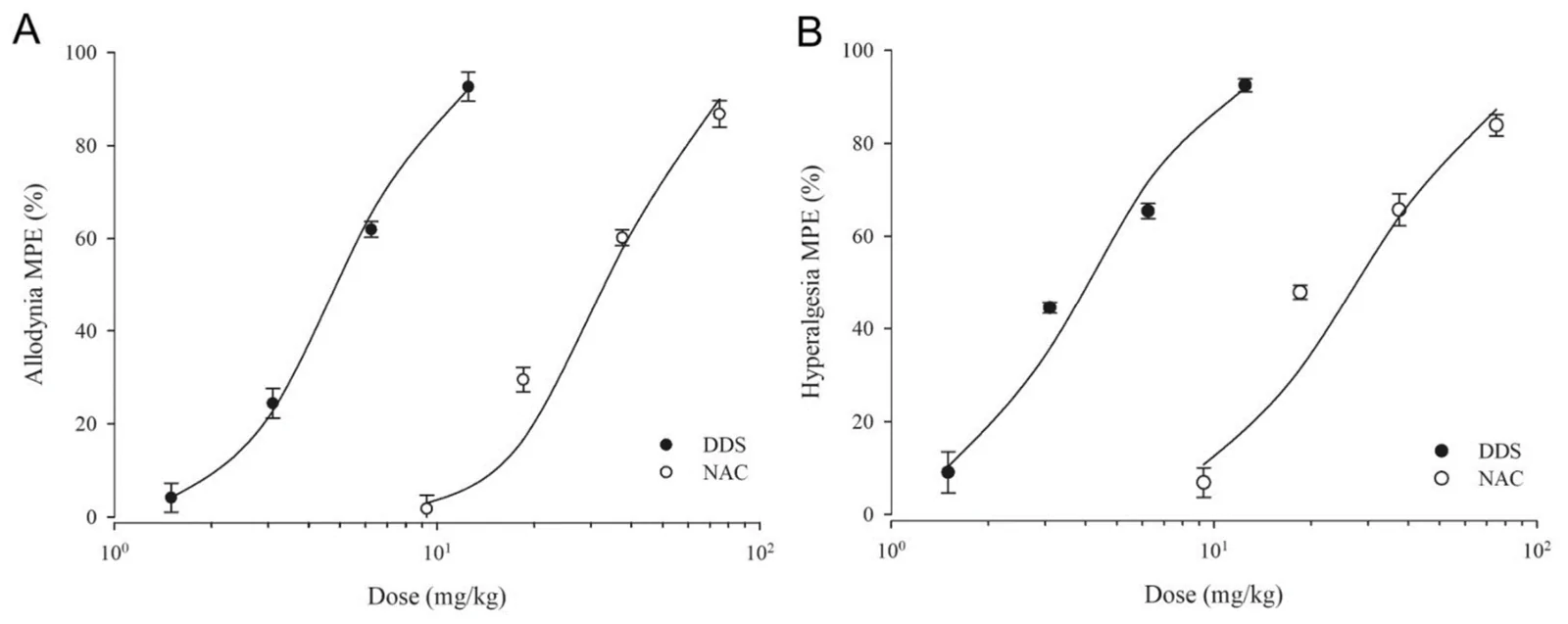
Panel (**A**) Individual dose–response curves of dapsone and N-acetylcysteine in mechanical allodynia. Effects are expressed as %MPE. Points represent mean observed responses ± S.E.M. (*n* = 6) and continuous lines represent nonlinear regression fits to a sigmoidal Emax/Hill model. DDS showed a leftward-shifted curve relative to NAC, consistent with greater pharmacological potency. Panel (**B**) Individual dose–response curves of dapsone and N-acetylcysteine in hyperalgesia. Effects are expressed as %MPE. Points represent mean observed responses ± S.E.M. (*n* = 6), and continuous lines represent nonlinear regression fits to a sigmoidal Emax/Hill model. DDS remained more potent than NAC, as reflected by the lower ED_50_.

The estimated $ED_{50}$ values were 4.92 mg/kg for DDS and 33.46 mg/kg for NAC, with Hill coefficients of 2.642 and 2.721, respectively. Model fit was adequate in both cases, with $R^{2}$ values of 0.977 for DDS and 0.889 for NAC, and RMSE values of 5.63 and 11.73, respectively. As summarized in Table 2 and illustrated in Figure 2A, both compounds produced dose-dependent antiallodynic effects with maximal efficacy approaching 100% MPE; however, the DDS curve was clearly shifted to the left relative to that of NAC, indicating greater pharmacological potency.

The potency ratio derived from the $ED_{50}$ values was 6.799, indicating that NAC required approximately 6.8-fold more dose than DDS to produce 50% of the effect.

A similar pattern was observed in hyperalgesia (Figure 2B). The DDS curve was described by the following:

$$E_{DDS-Hyp}=\frac{100\cdot D^{2.166}}{4.06^{2.166}+D^{2.166}}$$

whereas the NAC curve was fitted by the following:

$$E_{NAC-Hyp}=\frac{100\cdot D^{1.936}}{27.74^{1.936}+D^{1.936}}$$

The estimated $ED_{50}$ values were 4.06 mg/kg for DDS and 27.74 mg/kg for NAC, with Hill coefficients of 2.166 and 1.936, respectively. The corresponding $R^{2}$ values were 0.920 for DDS and 0.907 for NAC, whereas the RMSE values were 8.09 and 8.72, respectively. As shown in Figure 2 and summarized in Table 2, both compounds again reached high maximal efficacy, but DDS remained the more potent compound. The potency ratio calculated from the $ED_{50}$ values was 6.832, indicating that NAC required approximately 6.83-fold more dose than DDS to produce the same antihyperalgesic effect. The close similarity between the equipotent ratios obtained in both endpoints supported the use of a virtually common fixed-ratio design for interaction analysis.

For allodynia, the theoretical additive point corresponding to a 50% effect was located at 2.46 mg/kg DDS and 16.73 mg/kg NAC, with a $Z_{add}$ of 19.19 mg/kg. For hyperalgesia, the corresponding additive point was located at 2.03 mg/kg DDS and 13.87 mg/kg NAC, with a $Z_{add}$ of 15.90 mg/kg. These parameters provided the quantitative basis for evaluating the experimental mixture against the expectation of simple additivity.

**Figure 3:**
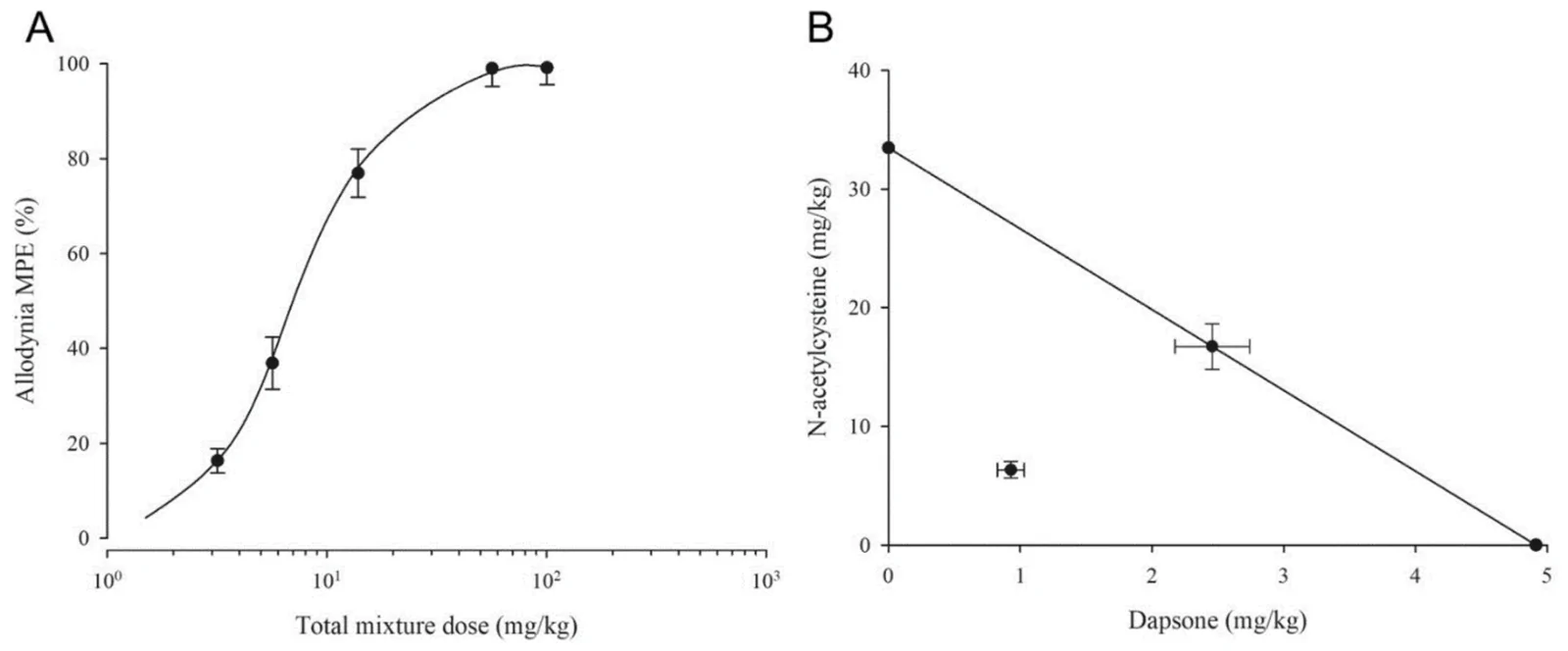
Panel (**A**) Dose–response curve of the DDS/NAC combination in mechanical allodynia. Effects are expressed as %MPE. Points represent mean responses ± S.E.M. (*n* = 6) at each total mixture dose, and the continuous line represents the nonlinear regression fit to a sigmoidal Emax/Hill model. The experimentally determined total ED50 was 7.45 mg/kg. Panel (**B**) Isobologram showing Dapsone and N-acetylcysteine synergy in mechanical allodynia. The line links their individual ED50 values, indicating additivity. The mixture’s ED_50_ is below this line, demonstrating synergy. Error bars reflect bootstrap uncertainty for the experimental point.

In allodynia, the mixture produced mean %MPE values of 16.33 ± 2.58, 36.87 ± 5.47, 76.94 ± 5.10, 99.00 ± 3.82, and 99.14 ± 3.56 at total doses of 3.18, 5.66, 13.90, 56.62, and 100.70 mg/kg, respectively. The naive, sham, and vehicle groups showed mean values of 107.70 ± 2.25, 100.00 ± 1.65, and 0.00 ± 0.92%MPE, respectively. One-way ANOVA revealed a highly significant treatment effect (F_7,40_ = 149.37, *p* = 1.29 × 10^−26^). Tukey’s *post hoc* test showed that all combination groups differed significantly from vehicle, including the lowest-dose mixture. The two highest combinations were not significantly different from sham or naive, and they were also not significantly different from each other, indicating that maximal antiallodynic efficacy had already been achieved at the 56.62 mg/kg total dose. In contrast, the third combination remained significantly lower than the fourth and fifth combinations, indicating that the ascending phase of the mixture dose–response curve was still discernible at that level. As shown in Figure 3A, the mixture produced a marked dose-dependent antiallodynic response with a clear sigmoidal profile.

Fitting of the allodynia combination data yielded the following equation:

$$E_{mix-Alo}\left(Z\right)=\frac{100\cdot Z^{1.9405}}{7.45^{1.9405}+Z^{1.9405}}$$

The experimental total ED_50_ was 7.45 mg/kg, and the Hill slope was 1.9405. As summarized in Table 3, the nonlinear fit also yielded derived doses of 3.65 mg/kg for ED_20_, 7.45 mg/kg for ED_50_, 15.21 mg/kg for ED_80_, and 23.11 mg/kg for ED_90_. When fit quality was assessed using individual-animal data, the model showed an R^2^ of 0.9259 and an RMSE of 9.49.

Bootstrap analysis confirmed the stability of the allodynia mixture fit. The median ED_50_ was 7.44 mg/kg (95% CI: 6.55–8.73), whereas the median ED_20_, ED_80_, and ED_90_ values were 3.64 mg/kg (95% CI: 3.05–4.41), 15.06 mg/kg (95% CI: 12.37–20.39), and 22.72 mg/kg (95% CI: 17.57–33.96), respectively. The bootstrap median Hill slope was 1.96 (95% CI: 1.55–2.42). These estimates, presented in Table 4, support the robustness of the fitted model.

The interaction index for allodynia was γ = 0.389, and bootstrap analysis yielded a 95% confidence interval of 0.341–0.455. Because this interval remained entirely below 1, the interaction was confirmed to be synergistic. In potency terms, the combination was 2.58-fold more potent than predicted by simple additivity. Projection of the experimental ED_50_ onto the theoretical equipotent ratio placed the experimental point at 0.955 mg/kg DDS and 6.49 mg/kg NAC, clearly below the theoretical additive point, as shown in Figure 3B. The main isobolographic parameters for this endpoint are summarized in Table 5.

**Figure 4:**
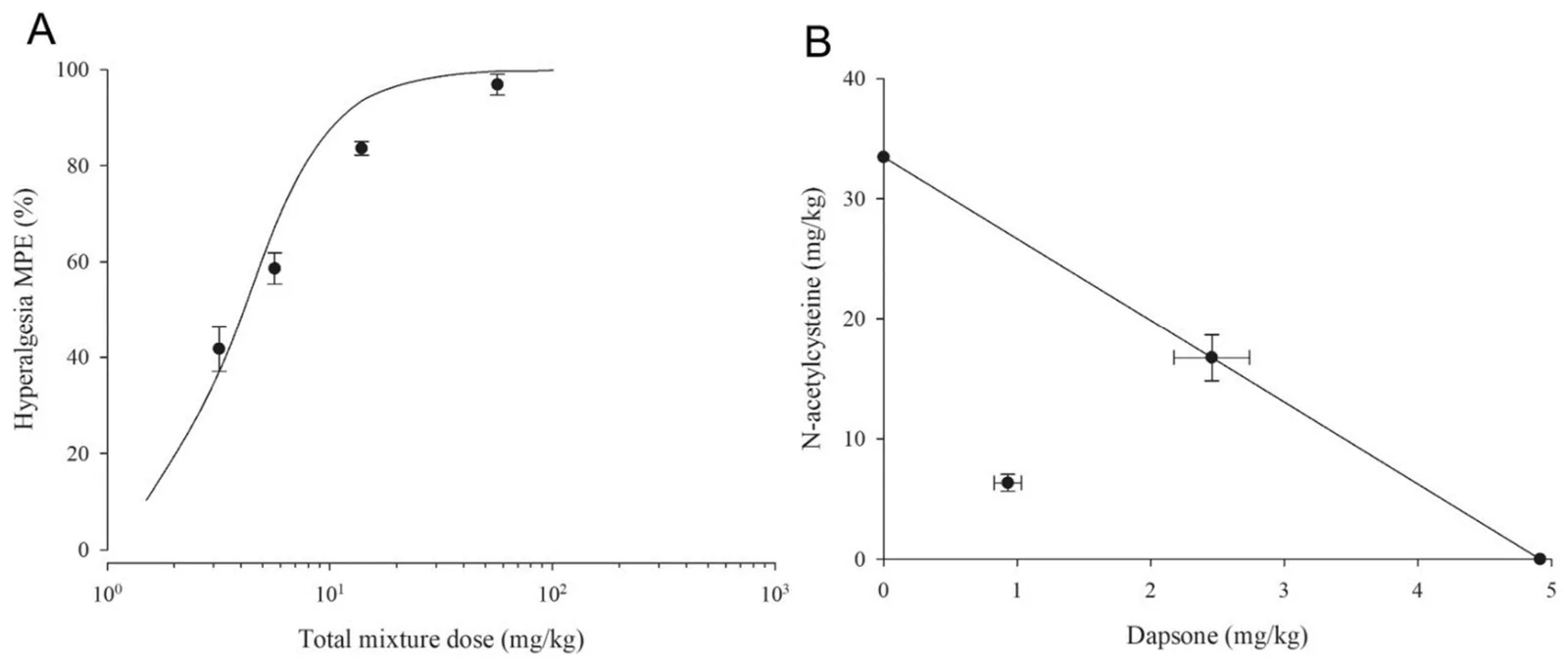
(**A**) Dose–response curve for the Dapsone/NAC combination in hyperalgesia. Each point shows the mean ± S.E.M. of %MPE at each total mixture dose (*n* = 6), with the continuous line representing a nonlinear regression fit to a sigmoidal Emax/Hill model based on average values. The experimentally determined total ED_50_ for the combination was 4.21 mg/kg (R^2^ = 0.9845, RMSE = 2.95). (**B**) Isobologram of Dapsone/NAC in hyperalgesia. The straight line shows calculated additivity from individual ED_50_ values. The mixture’s projected ED_50_ falls below this line, confirming synergy between the compounds. Error bars denote bootstrap-derived uncertainty for the experimental point.

In hyperalgesia, the same combination doses produced mean %MPE values of 41.75 ± 4.65, 58.59 ± 3.24, 83.59 ± 1.40, 96.87 ± 2.17, and 105.03 ± 1.59 at total doses of 3.18, 5.66, 13.90, 56.62, and 100.70 mg/kg, respectively. Naive, sham, and vehicle groups showed mean values of 106.05 ± 2.30, 100.00 ± 2.69, and 0.00 ± 2.19%MPE, respectively. One-way ANOVA again revealed a highly significant treatment effect (F_7,40_ = 152.48, *p* = 8.69 ×10^−27^), and Tukey’s post hoc test demonstrated that all combination groups differed significantly from vehicle. The two highest combinations were not significantly different from sham or naive, and the difference between the third and fourth combinations did not reach significance, suggesting that the mixture approached maximal antihyperalgesic efficacy at lower doses than in allodynia. As shown in Figure 4A, the combination produced a steep and highly effective dose-dependent antihyperalgesic response.

The hyperalgesia combination data were fitted by the following equation:

$$E_{mix-Hip}\left(Z\right)=\frac{100\cdot Z^{1.3747}}{4.21^{1.3747}+Z^{1.3747}}$$

The experimental total ED_50_ was 4.21 mg/kg, and the Hill slope was 1.3747. As shown in Table 3, the derived mixture doses were 1.54 mg/kg for ED_20_, 4.21 mg/kg for ED_50_, 11.54 mg/kg for ED_80_, and 20.81 mg/kg for ED_90_. When evaluated using individual-animal data, the fit yielded an R^2^ of 0.8924 and an RMSE of 8.16.

Bootstrap analysis confirmed the stability of the hyperalgesia mixture fit. The median ED_50_ was 4.16 mg/kg (95% CI: 3.49–5.00), whereas the median ED_20_, ED_80_, and ED_90_ values were 1.51 mg/kg (95% CI: 1.08–2.22), 11.50 mg/kg (95% CI: 9.95–13.32), and 20.85 mg/kg (95% CI: 16.12–26.42), respectively. The bootstrap median Hill slope was 1.36 (95% CI: 1.14–1.77). These results are presented in Table 4.

The interaction index in hyperalgesia was γ = 0.2647, with a bootstrap 95% confidence interval of 0.220–0.315, again entirely below 1 and therefore consistent with a robust synergistic interaction. In this endpoint, the combination was 3.78-fold more potent than expected from simple additivity. Projection of the experimental ED_50_ onto the theoretical equipotent ratio placed the experimental point at 0.5374 mg/kg DDS and 3.6720 mg/kg NAC, again below the theoretical additive point, as shown in Figure 4B. The complete isobolographic summary is provided in Table 5.

## 4. Discussion

Various studies have highlighted the therapeutic benefits of DDS, positioning it as a neuroprotective, safe, and effective treatment for various neurological diseases because of its ability to regulate different damage mechanisms, including oxidative stress, excitotoxicity, inflammation, and various cell death mechanisms [10]. On the other hand, NAC has been a target of study for pain treatment because oxidative stress is involved in the development and maintenance of the NP [33]. For this reason, the present study focused on evaluating the potential antinociceptive and antioxidant effects of combining DDS and NAC in rats with SCI. The SCI contusion model (T12, 6.25 mm) produced nociceptive responses characteristic of chronic NP (stimulus-dependent hypersensitivity) in the hind limbs of the study subjects starting from day 15 post-SCI, in agreement with a previous report by Mata-Bermudez et al. [34]. The results obtained suggest that subacute administration (once a day for 7 days) of DDS (1.5, 3.1, 6.25, and 12.5 mg/kg, i.p.) (Figure 1) can produce a dose-dependent antiallodynic and antihyperalgesic effect in rats with SCI, reaching values close to those obtained in the control groups (naïve and sham), and can even approach the values achieved by gabapentin (30 mg/kg, i.p.), a first-line drug used for the treatment of SCI-induced NP [35]. Similarly, a significant reduction in LP and an increase in GSH were observed compared with those in the group of animals that received only the vehicle as treatment. These results are consistent with those of previous reports indicating that subacute treatment (7 days) with DDS (12.5 mg/kg, i.p.) was able to reduce nociceptive behaviors (tactile allodynia and thermal hyperalgesia) in rats with vincristine-induced neuropathy; this effect was associated with a decrease in malondialdehyde levels and an increase in the amount of GSH present in the sciatic nerve [13].

Furthermore, another study reported that treatment with DDS (5, 10, and 20 mg/kg every 2 days for 2 weeks) decreased tactile allodynia, thermal hyperalgesia, and the levels of proinflammatory mediators (TNF-α, IL-1β, and NF-κB) in rats with osteoarthritis [36]. The antinociceptive effect induced by DDS in our experimental model can be attributed to its antioxidant properties and its ability to block oxidative stress mediated by the activation of NMDA receptors [37]. Oxidative stress is described as an injury event induced by the excessive production of ROS (e.g., superoxide anion, hydroxyl radical, and hydrogen peroxide) and a decrease in antioxidant systems to counteract them, resulting in cellular damage, especially in membrane phospholipids that are particularly vulnerable to ROS-mediated lipid peroxidation [38].

Additionally, subacute treatment (once daily for 7 days) with NAC (9.3, 18.7, 37.5, and 75 mg/kg, i.p.) (Figure 2) reduced tactile allodynia and mechanical hyperalgesia in a dose-dependent manner in rats with SCI (T12, 6.25 mm) through a reduction in LP and an increase in GSH levels in the injured tissue. The data obtained are consistent with those of various previous reports; for example, the administration of NAC (150 mg/kg/day, i.p.) inhibited nociceptive transmission in rats with chronic constriction of the sciatic nerve by decreasing the levels of LP and NO in the spinal cord [15,20]. Furthermore, another study revealed that NAC reduces mechanical allodynia and thermal hyperalgesia, effects that are accompanied by a significant reduction in the activity of superoxide dismutase 1 and 2, as well as LP products, in the plasma of rats with diabetic neuropathy [39]. Previous studies have reported that after traumatic spinal cord injury, an increase in LP is observed in the early and progressive stages of injury to the spinal cord, such as the appearance of polyunsaturated fatty acid peroxidation products (malondialdehyde), a decrease in cholesterol, and the appearance of cholesterol oxidation products [40,41]. Furthermore, a decrease in GSH and glutathione reductase levels in the spinal cord has been observed from the first hour post-injury and up to at least 48 h after injury [42]. In this same context, a significant increase in LP and a decrease in GSH levels in the NP state have been suggested [43,44]. In general, treatment with DDS and NAC, which are used as individual therapies, is promising because they reduce nociceptive behaviors and modulate oxidative stress in rats with SCI; however, the combination of both treatments considerably enhances these effects. Compared with the individual drug treatments, the combined DDS and NAC treatment produced a significantly greater antinociceptive effect (Figure 3). Additionally, we observed a significant improvement in oxidative stress markers (LP and GSH), suggesting a complementary antioxidant mechanism between the two drugs. In our study, the effective dose 50 (ED_50_) values obtained for the combined treatment were consistently below the additivity line (based on the calculation of the ED_50_ of each drug separately from their respective dose–response curves), which suggests that the interaction between DDS and NAC is synergistic. This type of interaction enables a reduction in individual doses, thereby decreasing the likelihood of adverse effects [45,46]. The drugs used exhibit diverse and complex mechanisms of action. DDS has demonstrated its antinociceptive capacity through the modulation of neuroinflammation and oxidative stress in different models of NP [11,13]. Furthermore, DDS may block NMDA receptors [12,37], especially those located in the dorsal horn of the spinal cord, which are associated with the generation and maintenance of the NP [47,48]. Moreover, the therapeutic efficacy of NAC for managing the NP in this experimental model is attributed to its antioxidant properties, as it is a cysteine prodrug and a precursor of glutathione (GSH) [49]. The simultaneous effect of DDS and NAC may be involved in regulating extracellular glutamate levels, potentially inhibiting excessive Ca^2+^ influx into neuronal terminals through the phosphorylation of NMDA receptors, thereby reducing excitotoxic damage, modulating nociceptive transmission, and influencing synaptic plasticity [50]. In support of this, the abnormal increase in neuronal responses is known to play a crucial role in the development of abnormal pain, known as central sensitization, which is closely related to the involvement of the NMDA receptor [51]. DDS is known to act as an antagonist of the neurodegenerative effects induced by NMDA agonists [12,37,52], whereas NAC is capable of modulating glutamatergic neurotransmission both directly and indirectly [53]. This hypothesis is supported by reports that blocking these receptors with various antagonists effectively suppressed tactile allodynia in animals with SCI [54], spinal cord ischemic injury, and sciatic nerve injury [55]. Furthermore, the NMDA receptor is permeable to Ca^2+^, which promotes depolarization of the terminals to activate voltage-dependent Ca^2+^ channels and indirectly triggers the release of neurotransmitters through intracellular signaling cascades independent of ion flow, thereby amplifying the nociceptive response [47,56]. In this context, spinal trauma produces a considerable increase in ROS at the injury site [57,58], where neurons are especially susceptible to damage induced by oxidative stress, contributing to the development of the NP [59]. Afferent discharges produced as a result of primary injury, followed by ectopic discharges in the spinal cord, can increase mitochondrial respiration as well as intracellular Ca^2+^ and consequently lead to an increase in ROS production [14]. It has been proposed that antioxidant agents produce antinociceptive effects by reducing central sensitization induced by peripheral nerve damage in animal models of NP, suggesting that ROS may be involved in central sensitization [60]. Therefore, DDS and NAC may decrease the flow of Ca^2+^ into the mitochondria, consequently reducing the formation of oxygen-free radicals [61] and NO [13], which explains the changes in OS markers (decrease in LP and increase in GSH concentration) in this experimental model.

Overall, these results demonstrate that DDS and NAC produce dose-dependent antinociceptive effects in both allodynia and hyperalgesia, with high maximal efficacy in both models, but with consistently greater potency for DDS. This interpretation is biologically plausible considering previous evidence showing that DDS has anti-inflammatory, antioxidant, and neuroprotective properties, and that it can attenuate allodynia and hyperalgesia in experimental neuropathic pain models [11,62,63]. Likewise, NAC has shown antiallodynic or antihyperalgesic effects in ischemia/reperfusion, chemotherapy-induced, and peripheral nerve injury models [64,65,66]. In addition, antioxidant-based combinations involving NAC have previously shown enhanced or synergistic analgesic effects in neuropathic pain-related conditions, which supports the pharmacological plausibility of the present interaction pattern [64,67].

Furthermore, it is worth mentioning that combination therapy provides evidence of its therapeutic utility. A meta-analysis by Mohiuddin et al. [68] reports that antihyperalgesic treatment with NAC alone is inconclusive, as it is only effective for certain types of pain. This is likely because it is necessary to combine medications with complementary therapeutic targets, as is the case with this therapy.

Finally, DDS has been shown to decrease NMDA receptor-mediated excitotoxicity, as well as reduce lipid peroxidation levels and increase GSH, resulting in a decrease in oxidative stress. Another mechanism related to the reduction in oxidative stress is the irreversible inhibition of myeloperoxidase, preventing the formation of hypochlorous acid. It also regulates the inflammatory response by decreasing the levels of various pro-inflammatory cytokines such as TNFα, IL-6, IL-8, IL-1β, etc. [10] and apoptosis observed lower activity of caspases 9, 8, and 3 in various conditions due to the effect of dapsone, demonstrating that it is a neuroprotective drug [58]; therefore, the downregulation of these damage mechanisms associated with hyperalgesia makes this therapy a good candidate. While treatment with NAC administered as monotherapy for pain has inconclusive results [68], since its main mechanism of action is as an antioxidant by increasing the effectiveness of antioxidant defenses, the combination of DDS and NAC may have more successful therapeutic effects.

Furthermore, it is important to mention that in this study we used safe dose ranges of DDS (1.5 to 12.5 mg/kg) and NAC (9.3 to 75 mg/kg), since the toxic levels reported in rats for DDS and NAC are greater than 25 mg/kg and 600 mg/kg, respectively [69,70]; therefore, no adverse or toxic effects were observed when the treatments were administered alone or in combination.

Other important information that demonstrates that this therapy is safe is that peripheral neuropathy is a rare event associated with the chronic use of DDS in humans. It commonly affects motor nerves, but also sensory pathways may be altered. In rats, however, this effect has not been reported [71]. On the contrary, DDS administration to rats prevented vincristine-induced neuropathy through anti-inflammatory, anti-oxidant, and anti-apoptotic mechanisms [13]. Taken together, these findings indicate that the DDS/NAC combination not only preserves the analgesic activity of both compounds but also potentiates it significantly beyond the simple sum of their individual effects, particularly in the hyperalgesic component.

## 5. Conclusions

Based on the results, this study demonstrated that treatment with DDS or NAC alone is capable of exerting antinociceptive activity in rats with SCI. These effects were modulated by the reduction in excitotoxicity associated with the activation of NMDA receptors. Furthermore, the combination of DDS and NAC has synergistic antinociceptive and antioxidant effects, as indicated by isobolographic analysis. These findings demonstrate the significant therapeutic potential of the DDS and NAC combination, suggesting that it is a novel, safe, and effective treatment for managing NP induced by SCI in patients. The limitation of this study is that it was conducted in an animal model of spinal cord injury; therefore, further studies will be required before the treatments can be used in clinical pain studies in human patients.

## 6. Limitations of the Study

Despite numerous reports describing the neuroprotective and antihyperalgesic mechanisms of DDS and NAC in various pathologies, this study only demonstrates the antihyperalgesic effect of the combination therapy associated with oxidative stress. Given the interest in this association and its regulatory properties, we propose future studies to characterize other markers such as enzymatic and antioxidant activities, markers of the inflammatory response, and apoptosis. Those studies will utilize molecular, electrophysiological, and histological techniques to understand and analyze the effect of combination therapy on the regulation of other mechanisms associated with the development of hyperalgesia following spinal cord injury. Furthermore, it would be important to corroborate these findings in male rats, since in this study and in other reports from our group, spinal cord injury is performed on female rats.

## Figures and Tables

**Table 1:** Doses of dapsone and *N*-acetylcysteine administered as monotherapies and as mixtures for the isobolographic analysis in rats with spinal cord injury.

Monotherapy Doses (mg/kg, i.p.)		Combination Doses Used in the Isobolographic Study (mg/kg, i.p.)		
DDS	NAC	DDS Component	NAC Component	Total Combination Dose
1.5	9.3	0.43	2.75	3.18
3.1	18.5	0.76	4.90	5.66
6.25	37.5	2.41	11.49	13.90
12.5	75	7.63	48.99	56.62
		13.58	87.12	100.70

**Note:** DDS, dapsone; NAC, *N*-acetylcysteine; i.p., intraperitoneal; SCI, spinal cord injury. SCI was induced at the T12 level using a 6.25 mm impact height. The total mixture dose is the sum of the DDS and NAC components. Blank cells indicate that no additional monotherapy dose was evaluated.

**Table 2:** Pharmacometric parameters estimated from the individual dose–response curves of dapsone and N-acetylcysteine for mechanical allodynia and hyperalgesia.

Outcome	Compound	Potency Parameters		Model Fit		Comparative Potency
		ED50 (mg/kg)	Hill Slope	R^2^	RMSE (%MPE)	ED_50_ Ratio (NAC/DDS)
Allodynia	DDS	4.92	2.642	0.977	5.63	6.799
	NAC	33.46	2.721	0.889	11.73	
Hyperalgesia	DDS	4.06	2.166	0.920	8.09	6.832
	NAC	27.74	1.936	0.907	8.72	

**Note:** Parameters were estimated by nonlinear regression using a sigmoidal Emax model. ED50, the dose producing 50% of the maximum possible effect; R^2^, coefficient of determination; RMSE, root mean square error; %MPE, percentage of maximum possible effect. The potency ratio was calculated as ED50,NAC/ED50,DDS from the unrounded estimates; values greater than 1 indicate greater potency of DDS relative to NAC. *n* = 6 per experimental group.

**Table 3:** Pharmacometric parameters for the antiallodynic and antihyperalgesic effects of the fixed-ratio dapsone (DDS)/N-acetylcysteine (NAC) mixture estimated by nonlinear regression.

Behavioral Endpoint	ED_20_ (mg/kg)	ED_50_ (mg/kg)	ED_80_ (mg/kg)	ED_90_ (mg/kg)	Hill Slope	Fit to Mean Responses		Fit to Individual Observations	
						R^2^	RMSE (%MPE)	R^2^	RMSE (%MPE)
Allodynia	3.65	7.45	15.21	23.11	1.9405	0.9998	0.44	0.9259	9.49
Hyperalgesia	1.54	4.21	11.54	20.81	1.3747	0.9845	2.95	0.8924	8.16

**Note:** ED_x_, dose producing x% of the maximum effect; %MPE, percentage of the maximum possible effect; R^2^, coefficient of determination; RMSE, root-mean-square error. Mean-response fits were based on group means (*n* = 6 per experimental group).

**Table 4:** Bootstrap estimates and 95% confidence intervals for the pharmacometric parameters of the fixed-ratio dapsone (DDS)/N-acetylcysteine (NAC) mixture.

Behavioral Endpoint	Parameter	Bootstrap Median	95% CI
Allodynia	ED_20_ (mg/kg)	3.64	3.05–4.41
	ED_50_ (mg/kg)	7.44	6.55–8.73
	ED_80_ (mg/kg)	15.06	12.37–20.39
	ED_90_ (mg/kg)	22.72	17.57–33.96
	Hill slope	1.96	1.55–2.42
	γ	0.389	0.341–0.455
Hyperalgesia	ED_20_ (mg/kg)	1.51	1.08–2.22
	ED_50_ (mg/kg)	4.16	3.49–5.00
	ED_80_ (mg/kg)	11.50	9.95–13.32
	ED_90_ (mg/kg)	20.85	16.12–26.42
	Hill slope	1.36	1.14–1.77
	γ	0.265	0.220–0.315

**Note:** CI, confidence interval; ED_x_, dose producing x% of the maximum effect; γ, interaction index calculated as *Z*_mix_/*Z*_add_ (γ < 1 indicates synergism, γ = 1 additivity, and γ > 1 antagonism).

**Table 5:** Isobolographic analysis of the fixed-ratio dapsone (DDS)/*N*-acetylcysteine (NAC) mixture.

Outcome	Individual-Drug ED_50_ (mg/kg)		Theoretical Additive Dose	Experimental Mixture Dose	Interaction Metrics		Components of Zmix (mg/kg)	
	DDS	NAC	Zadd (mg/kg)	Zmix (mg/kg)	γ = Zmix/Zadd	Potency Shift Zadd/Zmix	DDS Component	NAC Component
Allodynia	4.92	33.47	19.19	7.45	0.388	2.58	0.955	6.492
Hyperalgesia	4.06	27.74	15.90	4.21	0.265	3.78	0.537	3.672

**Note:** ED_50_, the dose producing 50% of the maximum possible effect; Zadd, theoretical additive ED_50_; Zmix, experimentally determined ED_50_ of the mixture; γ, interaction index calculated as Zmix/Zadd; potency shift, Zadd/Zmix. A γ value below 1 indicates synergism. The 1:1 fixed ratio denotes equal fractions of the individual-drug ED_50_ values rather than equal mass doses. Component doses sum to Zmix; minor discrepancies may arise from rounding.

## Data Availability

The dataset is available on request from the authors.

## Supplementary Materials

The following supporting information can be downloaded at: https://www.mdpi.com/article/10.3390/brainsci16080777/s1, Table S1: The ARRIVE guidelines 2.0: author checklist.
