# Supplementary material for: Dapsone and N-Acetylcysteine Exert a Synergistic Antinociceptive Effect Associated with Oxidative Stress After Traumatic Spinal Cord Injury: An Isobolographic Study
Source: Brain Sci. 2026 Jul 23;16(8):777. doi: 10.3390/brainsci16080777 (PMC13511174; doi:10.3390/brainsci16080777)
Supplement: Supplementary file 1 [file brainsci-16-00777-s001.zip › brainsci-4401387-supplementary.pdf]

NOTE: Please save this file locally before filling in the table, DO NOT work on the file within your internet browser as changes will not be saved. Adobe Acrobat Reader (available free here) is recommended for completion.

The ARRIVE guidelines 2.0: author checklist

The ARRIVE Essential 10

These items are the basic minimum to include in a manuscript. Without this information, readers and reviewers cannot assess the reliability of the findings.

Item		Recommendation	Section/line number, or reason for not reporting
**Study design**	1	For each experiment, provide brief details of study design including:	
		a. The groups being compared, including control groups. If no control group has been used, the rationale should be stated.	Yes , the groups being compared, including control groups. In Experimental design secction
		b. The experimental unit (e.g. a single animal, litter, or cage of animals).	Animal (rat)
**Sample size**	2	a. Specify the exact number of experimental units allocated to each group, and the total number in each experiment. Also indicate the total number of animals used.	126 animals were used Page 7 lines 3 to 7 page 11, lines 9 to 16.
		b. Explain how the sample size was decided. Provide details of any *a priori* sample size calculation, if done.	Page 11 lines 15-22 and page 12, lines 1-13
**Inclusion and exclusion criteria**	3	a. Describe any criteria used for including and excluding animals (or experimental units) during the experiment, and data points during the analysis. Specify if these criteria were established *a priori*. If no criteria were set, state this explicitly.	Page 9, lines 19 and 20
		b. For each experimental group, report any animals, experimental units or data points not included in the analysis and explain why. If there were no exclusions, state so.	Page 9, lines 19 and 20
		c. For each analysis, report the exact value of *n* in each experimental group.	The number of animals used is reported in the figure captions.
**Randomisation**	4	a. State whether randomisation was used to allocate experimental units to control and treatment groups. If done, provide the method used to generate the randomisation sequence.	Page 11 lines 14-22
		b. Describe the strategy used to minimise potential confounders such as the order of treatments and measurements, or animal/cage location. If confounders were not controlled, state this explicitly.	The strategy was the randomization of the treatments to be applied to the animals. A second control was tthe blinding of the observation to the treatment asignement Page12, lines 12 and 13
**Blinding**	5	Describe who was aware of the group allocation at the different stages of the experiment (during the allocation, the conduct of the experiment, the outcome assessment, and the data analysis).	Araceli Diaz-Ruiz, Alfonso Mata-Bermudez, and Héctor Alonso Romero-Sánchez.
**Outcome measures**	6	a. Clearly define all outcome measures assessed (e.g. cell death, molecular markers, or behavioural changes).	Markers of behavioral alterations (hyperalgesia) and biochemical markers of damage from oxidative stress
		b. For hypothesis-testing studies, specify the primary outcome measure, i.e. the outcome measure that was used to determine the sample size.	The calculation of the sample size depends on the variability of the methods. See Page 11 lines 16-22.
**Statistical methods**	7	a. Provide details of the statistical methods used for each analysis, including software used.	Page 14, lines 1 and Page 15 lines 1-21
		b. Describe any methods used to assess whether the data met the assumptions of the statistical approach, and what was done if the assumptions were not met.	Page 14, lines 1 and Page 15 lines 1-7
**Experimental animals**	8	a. Provide species-appropriate details of the animals used, including species, strain and substrain, sex, age or developmental stage, and, if relevant, weight.	Page 7 lines 3 and 4
		b. Provide further relevant information on the provenance of animals, health/immune status, genetic modification status, genotype, and any previous procedures.	Page 7, lines 7-14
**Experimental procedures**	9	For each experimental group, including controls, describe the procedures in enough detail to allow others to replicate them, including:	
		a. What was done, how it was done and what was used.	All the required information is in the methods section
		b. When and how often.	All the required information is in the methods section
		c. Where (including detail of any acclimatisation periods).	All the required information is in the methods section
		d. Why (provide rationale for procedures).	All the required information is in the methods section
**Results**	10	For each experiment conducted, including independent replications, report:	
		a. Summary/descriptive statistics for each experimental group, with a measure of variability where applicable (e.g. mean and SD, or median and range).	All the information can be found in the results section.
		b. If applicable, the effect size with a confidence interval.	All the information can be found in the results section.

The Recommended Set

These items complement the Essential 10 and add important context to the study. Reporting the items in both sets represents best practice.

Item		Recommendation	Section/line number, or reason for not reporting
**Abstract**	11	Provide an accurate summary of the research objectives, animal species, strain and sex, key methods, principal findings, and study conclusions.	Page 3 lines 2-23
**Background**	12	a. Include sufficient scientific background to understand the rationale and context for the study, and explain the experimental approach.	Yes, this information is contained in the introduction section.
		b. Explain how the animal species and model used address the scientific objectives and, where appropriate, the relevance to human biology.	In translational medicine studies, the use of animals is useful to demonstrate that new drug therapies are safe and effective.
**Objectives**	13	Clearly describe the research question, research objectives and, where appropriate, specific hypotheses being tested.	Treatment with dapsone and NAC, administere
**Ethical statement**	14	Provide the name of the ethical review committee or equivalent that has approved the use of animals in this study, and any relevant licence or protocol numbers (if applicable). If ethical approval was not sought or granted, provide a justification.	The information can be found in the animals section, page 7, lines 1-14.
**Housing and husbandry**	15	Provide details of housing and husbandry conditions, including any environmental enrichment.	The information can be found in the animals se
**Animal care and monitoring**	16	a. Describe any interventions or steps taken in the experimental protocols to reduce pain, suffering and distress.	Page 8 lines 4-8
		b. Report any expected or unexpected adverse events.	The drugs used in this study are repurposed and are used in humans for other conditions, so no serious adverse effects that put the animals' lives at risk have been reported.
		c. Describe the humane endpoints established for the study, the signs that were monitored and the frequency of monitoring. If the study did not have humane endpoints, state this.	In this study, a stimulus-dependent hyperalgesia model was used, so the animals only feel pain when the stimulus is applied, therefore the suffering is moderate.
**Interpretation/ scientific implications**	17	a. Interpret the results, taking into account the study objectives and hypotheses, current theory and other relevant studies in the literature.	The results were interpreted with the support of reports in the literature, as shown in the discussion.
		b. Comment on the study limitations including potential sources of bias, limitations of the animal model, and imprecision associated with the results.	The limitation of this study is that it was conducted in an animal model of spinal cord injury; therefore, further studies will be required before the treatments can be used in clinical pain studies in human patients.
**Generalisability/ translation**	18	Comment on whether, and how, the findings of this study are likely to generalise to other species or experimental conditions, including any relevance to human biology (where appropriate).	Based on the promising findings, as well as the therapeutic safety of the drugs (dapsone and N-acetylcysteine), which are currently used in humans for other conditions and have FDA approval, these drugs have high therapeutic potential for use in clinical trials.
**Protocol registration**	19	Provide a statement indicating whether a protocol (including the research question, key design features, and analysis plan) was prepared before the study, and if and where this protocol was registered.	In page 7, lines 7-14
**Data access**	20	Provide a statement describing if and where study data are available.	In page 32, line 21 and page 33, line 1
**Declaration of interests**	21	a. Declare any potential conflicts of interest, including financial and non-financial. If none exist, this should be stated.	In page 32, lines 17-20
		b. List all funding sources (including grant identifier) and the role of the funder(s) in the design, analysis and reporting of the study.	In page 32, lines 12-16
